# Prediction and identification of novel HLA-A*0201-restricted cytotoxic T lymphocyte epitopes from endocan

**DOI:** 10.1186/s12950-020-00240-w

**Published:** 2020-02-19

**Authors:** Gaohai Shao, Qingjun Liu, Ling Yang, Guibo Feng, Wang Zhao, Zhongyan Huang, Zhao Yang

**Affiliations:** 1 Department of orthopedics, Yongchuan Hospital, Chongqing Medical University, Chongqing, 402160 China; 2 Department of Neurology and Chongqing key laboratory of cerebravascular disease, Yongchuan Hospital, Chongqing Medical University, Chongqing, 402160 China

**Keywords:** HLA-A*0201, Cytotoxic T lymphocyte, Epitopes, Endocan

## Abstract

**Background:**

Prediction and identification of cytotoxic T lymphocyte (CTL) epitopes from tumor associated antigens is a crucial step for the development of tumor immunotherapy strategy. Endocan has been identified as antigen overexpressed in various tumors.

**Methods:**

In this experiment, we predicted and identified HLA-A2-restricted CTL epitopes from endocan by using the following procedures. Firstly, we predicted the epitopes from the amino acid sequence of endocan by computer-based methods; Secondly, we determined the affinity of the predicted peptide with HLA-A2.1 molecule by peptide-binding assay; Thirdly, we elicited the primary T cell response against the predicted peptides in vitro; Lastly, we tested the specific CTLs toward endocan and HLA-A2.1 positive target cells.

**Results:**

These data demonstrated that peptides of endocan containing residues 4–12 and 9–17 could elicit specific CTLs producing interferon-γ and cytotoxicity.

**Conclusions:**

Therefore, our findings suggested that the predicted peptides were novel HLA-A2.1-restricted CTL epitopes, and might provide promising target for tumor immunotherapy.

## Introduction

Gliomas are the most common primary central nervous system tumors [1–3]. Glioblastomas represent 50% of all gliomas in adults with an extremely poor prognosis. Despite innovative therapeutic methods such as surgical resection, chemotherapy, and radiation therapy, the survival ratio is very low [4–6]. Therefore, the novel therapeutic approaches are in great needed.

Endocan is a soluble proteoglycan of 50 kDa, consisting of a mature polypeptide of 165 amino acids and a single dermatan sulfate chain [7]. Endocan was originally described as being secreted by human umbilical vein endothelial cells (HUVECs) [8]. Recently, endocan has been identified to be upregulated in various tumors [9–11]. In addition, endocan has also been found in common brain tumors and acted as a marker for malignancy [12–14]. In recent years, endocan has been reported as an important biomarker for inflammation [15]. The related results confirm the hypothesis that human endocan may have a protective effect against acute lung inflammation [16].

Cellular adoptive immunotherapy has been widely utilized in the tumor therapy [17]. Tumor-associated antigens (TAA) represent promising targets for anticancer immunotherapies [18]. Peptides derived from TAAs are presented on the surface of tumor cells in association with MHC class I (MHC-I) complexes [19]. These peptide-MHC-I (pMHC-I) complexes can generate CTL mediated antitumor responses [20].

To date, endocan related CTL epitopes have not been identified. In this experiment, HLA-A*0201-restricted cytotoxic T lymphocyte epitopes from endocan were predicted, and the potential of generating CTL responses were analyzed.

## Materials and methods

### Cell lines and animals

The human TAP-deficient T2 cell line, BB7.2 cell line producing mAb against HLA-A2, human glioma cell line U251 (HLA-A2+), microglia cell line BV2 (HLA-A2-) were purchased from the American Type Culture Collection (Manassas, VA). Cells were cultured in RPMI-1640 medium containing 10% FBS (Gibco, with endotoxin level < 10 IU/ml), penicillin (200 U/ml), and streptomycin (100 μg/ml). All cell lines mentioned previously were kept at 37 °C in a humidified atmosphere containing 5% CO_2_. HLA-A*0201/Kb transgenic (Tg) mice, 8–12 weeks-old, were purchased from The Jackson Laboratory (USA). Mice were bred and maintained in specific pathogen-free (SPF) facilities. Animal experiments were performed in accordance with the guidelines of the Animal Care and Use Committee of Chongqing Medical University.

### Epitope prediction and synthesizing

A panel of endocan specific peptides (9 mer) containing HLA-A* 0201-binding motifs were predicted by software BIMAS (Section of BioInformatics and Molecular Analysis, National Institutes of Health, Bethesda, MD, USA) and SYFPEITHI (Institute for Immunology, University of Tübingen, Tübingen, Germany). The two 9-mer-peptides that demonstrated the highest scores with both SYFPEITHI and BIMAS were utilized for further analysis. Peptides were synthesized by F-moc chemistry. Before use, HPLC-purified peptides (> 95% pure) were dissolved at 40 mg/ml in DMSO and diluted to 1 mg/ml with Iscove’s modified Dulbecco’s medium (IMDM) (Life Technologies, Grand Island, NY).

### Peptide-binding assay

HLA-A*0201 Ag-processing defective T2 cell line was cultured in RPMI 1640 containing 10% FBS. Before use, the cells were incubated for 6 h at 37 °C in serum-free IMDM. Then, cells were washed once, suspended in serum-free IMDM containing 20 μM of 2-ME and 15 μg/ml of human β2-microglobulin (β2m) (Calbiochem, La Jolla, CA), and pulsed with 50 μM peptide. HLA-A2.1-restricted MAGE-2 CTL epitope KMVELVHFL (amino acid position in MAGE-2; 112–120) and Kb-restricted Hpa CTL epitope FSYGFFVI (amino acid position in Hpa; 519–526) served as positive and negative controls. After a 24 h incubation at 37 °C, T2 cells were washed once with cold PBS containing 0.5% BSA and 0.02% NaN_3_. They were then stained directly with primary anti-HLA-A2 Ab derived from BB7.2 and FITC-labeled goat-antimouse IgG (BD Biosciences Pharmingen, USA) secondary antibody. The percentage of FITC-positive cells as well as their staining intensity (mean fluorescence intensity (MFI)) was determined on an Epics Profile II (Coulter, Hialeah, FL). The Δ MFI for a particular mAb was calculated by subtracting the MFI of either the isotype-matched control mAb or the second-step Ab from each MFI value. The fluorescence ratio (FR) was calculated using the following formula: FR = (Δ MFI of peptide-treated T2 cells)/(Δ MFI of nontreated T2 cells).

### RT-PCR analysis of endocan expression

The tumor cells were homogenized using RNAiso Plus (Takara) and ceramic beads for 1 min in a speedmill plus according to the instructions of the manufacturer (Alytik Jena). RNA was isolated according to the instructions of the manufacturer and reverse transcripted to obtain cDNA using a PrimeScript™ RT Reagent Kit with gDNA Eraser (Takara). Real-time PCR was performed using cDNA samples with SYBR@Premix ExTaq™II (Takara, Tli RNaseH Plus) by the One-step Plus analyzer (ABI). We normalized the results for each individual gene using the housekeeping gene beta-actin. RT-PCR products were then run on a gel and visualized with ethidium bromide.

### Western blot analysis of endocan expression

Proteins from cultured tumor cells were resolved using SDS-PAGE and transferred onto polyvinylidene fluoride membranes using electroblotting. The membranes were incubated with primary antibodies, all diluted to 1:1000 (Cell Signaling Technology), at 4 °C overnight. β-actin (1:200; Santa Cruz Biotechnology, Dallas, TX) was used as the loading control. The membranes were incubated with HRP-conjugated goat anti-rabbit secondary Abs (1:2500; Sigma-Aldrich, St. Louis, MO) at 25 °C for 1 h. Bound Abs were visualized using a chemiluminescence detection system. Protein levels were calculated as the ratio of the target protein value to the β-actin value.

### Dendritic cell generation from human peripheral blood precursors

In brief, PBMC were isolated from HLA-A0201+ donors by ficoll-hypaque density gradient centrifugation. The cells were allowed to adhere in culture flasks for 2 h at 37 °C in RPMI1640 with 10% FBS. Then, non-adherent cells were collected and frozen in freezing media (60% RPMI-1640, 30% FBS and 10% DMSO) for later use in CTL assays. Adherent cells were cultured in 6 mL of RPMI-1640 with 10% FBS containing 800 U/mL recombinant human granulocyte-macrophage colony- stimulating factor (rhGM-CSF, R&D Systems, McKinley Place NE, MN, USA) and 1200 U/mL recombinant human interleukin-4 (rhIL-4, R&D Systems). On days 3, 5 and 7, half of the media was refreshed without discarding any cells and fresh cytokine-containing (rhGM-CSF and rhIL-4) media was added. On day 8 of culture, 1000 U/mL of tumor necrosis factor-a (TNF-a, R&D Systems) was added to the media. On day 9, non-adherent cells obtained from these cultures were considered mature human PBMC-derived DC.

### Induction of peptide-specific CTL with synthetic peptides

Mature human PBMC-derived DCs were pulsed with 100 lg/ml HLA-A0201- restricted CTL epitopes of endocan for 4 h. The DCs were then irradiated with 20 Gy to prevent outgrowth. Subsequently, the non-adherent autologous peripheral blood lymphocytes were co-cultured with the DC. After stimulation, 800 U/mL recombinant interleukin-2 (IL-2) was added. On day 7, and weekly thereafter, the autologous peripheral blood lymphocytes were restimulated with peptide-pulsed pulsed irradiated dendritic cells.

### ELISPOT assay

ELISPOT assay were utilized to analyze the inferferon-gamma (IFN-γ) production of CTLs. Briefly, effectors were plated in triplicate at a final concentration of 1 × 10^5^ cells/well in 96-well nitrocellulose plates. Effector cells were stimulated with candidate peptides at a final concentration of 30 μM. The plate was incubated at 37 °C, 5% CO_2_ for 24 h. The plate was processed using a biotin labeled anti-mouse IFN-γ antibody, an enzyme labeling marker and an antimarker. Then, a freshly prepared developer was added and incubated in the dark at 37 °C for 8 min (Quick Spot Mouse IFN-γ Precoated ELISPOT kit, Dakewe). Spots were quantified using the ELISPOT reader (BioReader 4000 Pro-X, BIOSYS, Germany).

### Cytotoxicity assay

To evaluate levels of CTL activity, a standard 4-h ^51^ Cr-release assay was used. In the autologous lymphocytes lysis assay, the autologous lymphocytes activated by PHA were used as target cells. Briefly, target cells were incubated with ^51^ Cr (100 μCi per 1× 10^6^ cells; Amersham Biosciences Corp) for 2 h in a 37 °C water bath. After incubation with ^51^Cr, target cells were washed three times with PBS, resuspended in RPMI 1640, and mixed with effector cells at effector-to-target (E/T) ratios of 25:1, 50:1, or 100:1. Assays were performed in triplicate wells for each experiment at each ratio in a 96-well round-bottomed plate. After a 4-h incubation, the supernatants were harvested, and the amount of ^51^Cr released was measured with a γ-counter. The percentage of specific lysate was calculated as 100 × (experimental release - spontaneous release) / (maximum release - spontaneous release). Maximum release was determined from supernatants of cells that were lysed by the addition of 2% Triton X-100.

### Analysis of in vivo immunogenicity

12 weeks old HLA-A*0201/Kb mice were immunized three times once a week, by subcutaneous injection in the back with 1 × 10^6^ syngeneic mature DC pulsed with the peptides. After 7 days, the spleens of the mice were removed and the splenocytes were harvested as effectors.

### Statistical analysis

All the experiments were done in triplicate, and the results are given as Means ± S.E.M. of triplicate determinations. Statistical analyses were performed using Student’s t-test. The difference was considered statistically significant when the *P* value was < 0.05.

## Results

Prediction of HLA-A*0201 restricted CTL epitopes of endocan.

To predict the HLA-A*0201-restricted CTL epitopes of endocan, we utilized two programs (BIMAS and SYFPEITHI) to analyze the total amino acid sequence of the protein. Top four 9-amino-acid peptides with highest scores were used as candidates for further analysis (Table 1). These peptides were chemically synthesized, purified, and identified. The molecular weight and the purity (> 95%) of each peptide were determined by mass spectrometry assay and HPLC.

**Table 1:** Predicted endocan epitopes binding to HLA-A2.1

Position	Length	Sequence	BIMAS score	SYFPEITHI score
4–12	9	VLLLTTLLV	437.482	26
9–17	9	TLLVPAHLV	257.342	24
6–14	9	LLTTLLVPA	19.425	22
3–11	9	SVLLLTTLL	6.916	21

### MHC peptide-binding assay

The high affinity peptides binding to HLA-A2.1 could enhance HLA-A2.1 expression of antigen processing-deficient T2 cells. Therefore, the binding affinity of the predicted peptides to HLA-A2.1 was determined by using antigen processing- deficient T2 cells. As shown in Table 2, the four peptides were bound to HLA-A2.1 molecules with various affinities. Of four peptides selected, endocan_**4–12**_ and endocan_**9–17**_ enhanced the HLA-A2.1 molecular expression and demonstrated high affinity to HLA-A2.1 molecule. However, endocan_**6–14**_ and endocan_**3–11**_ demonstrated low affinity to HLA-A2.1 molecule.

**Table 2:** HLA-A2-binding affinity of peptides

Name	Position	Length	Sequence	FI
endocan_4–12_	4–12	9	VLLLTTLLV	1.81
endocan_9–17_	9–17	9	TLLVPAHLV	1.76
endocan_6–14_	6–14	9	LLTTLLVPA	0.49
endocan_3–11_	3–11	9	SVLLLTTLL	0.43
MAGE-2	112–120	9	KMVELVHFL	1.76
Hpa _519–526_	519–526	8	FSYGFFVI	0.34

### Endocan mRNA and protein analysis

**Fig. 1:**
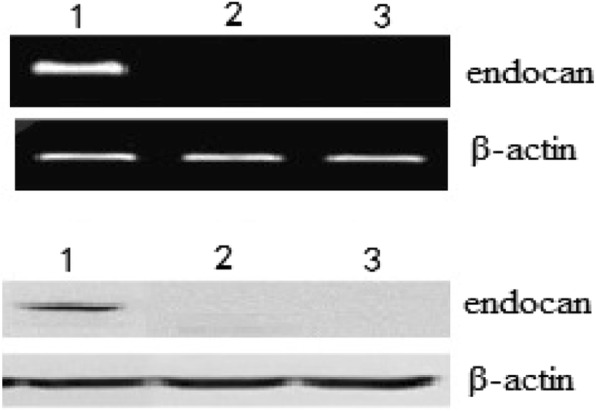
Endocan expression in different target cells. The tumor cells were homogenized, and total RNA was isolated using Tripure Isolation Regent Kit. Two microliters RT product was amplified with PCR by using TaqDNA polymerase (using standard procedures). RT-PCR products were then run on a gel and visualized with ethidium bromide. For Western blot analysis, proteins in the cell extracts were separated by SDS-PAGE and were then analyzed with anti- endocan MAb. 1: U251 cells; 2: BV2 cells; 3: autologous lymphocytes

To explore endocan levels of target cells, we analyzed endocan mRNA and protein in several cell lines by RT-PCR and Western blot assays. As shown in Fig. 1, endocan mRNA and protein could be detected in U251 cell lines. However, endocan mRNA and protein could not be detected in BV2 cell line and autologous lymphocytes.

### IFN-γ production analysis

**Fig. 2:**
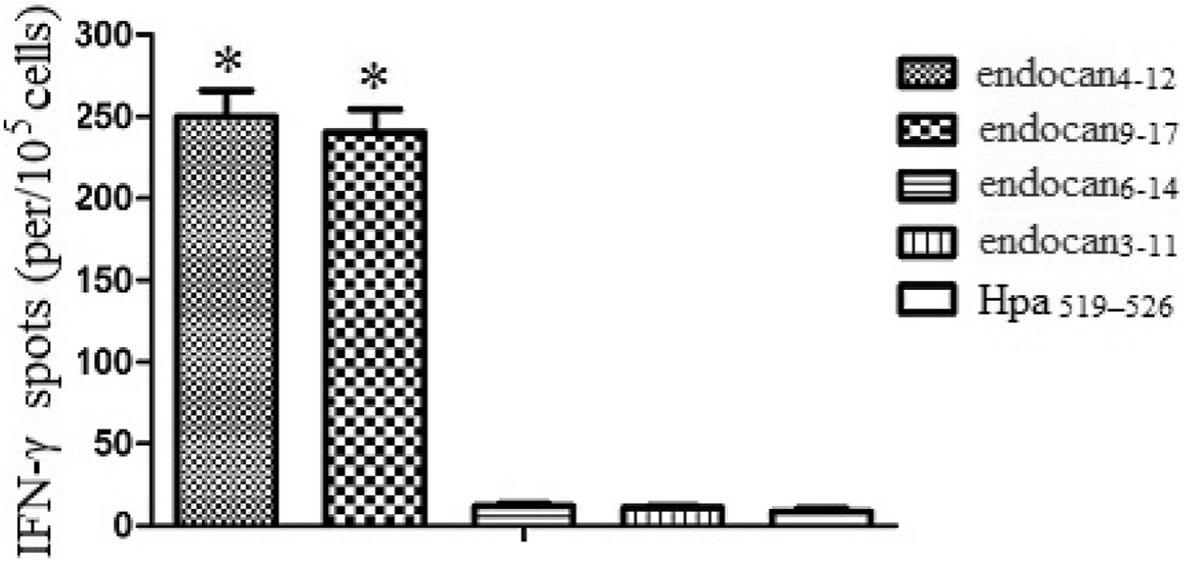
ELISPOT assay The PBMCs of human HLA-A2+ donors were obtained and then cultured in RPMI 1640 supplemented with 10% FCS, 100 U/ml penicillin, and 100 μg/ml streptomycin. DCs were generated, and loaded with different peptides at a final concentration of 100 μg/ml for 4 h and were then irradiated with 20 Gy, which prevented all outgrowths in the control cultures. Autologous T cells were restimulated every 7 days with the peptide-pulsed DCs to generate peptide-specific CTLs. The IFN-γ secretion was then assessed on day 23. Experiments performed in triplicate showed consistent results. Compared with controls, *P* < 0.05.

To detect whether predicted peptides could elicit CTL to produce IFN-γ, we analyzed IFN-γ-producing cells by ELISPOT assay. As shown in Fig. 2, endocan_**4–12**_ and endocan_**9–17**_ could elicit IFN-γ-producing cells. However, IFN-γ-producing cells could not be detected in endocan_**6–14**_ and endocan_**3–11**_ groups. These results suggested that.

endocan_**4–12**_ and endocan_**9–17**_ could effectively promote CTL response.

### Cytotoxicity analysis

**Fig. 3:**
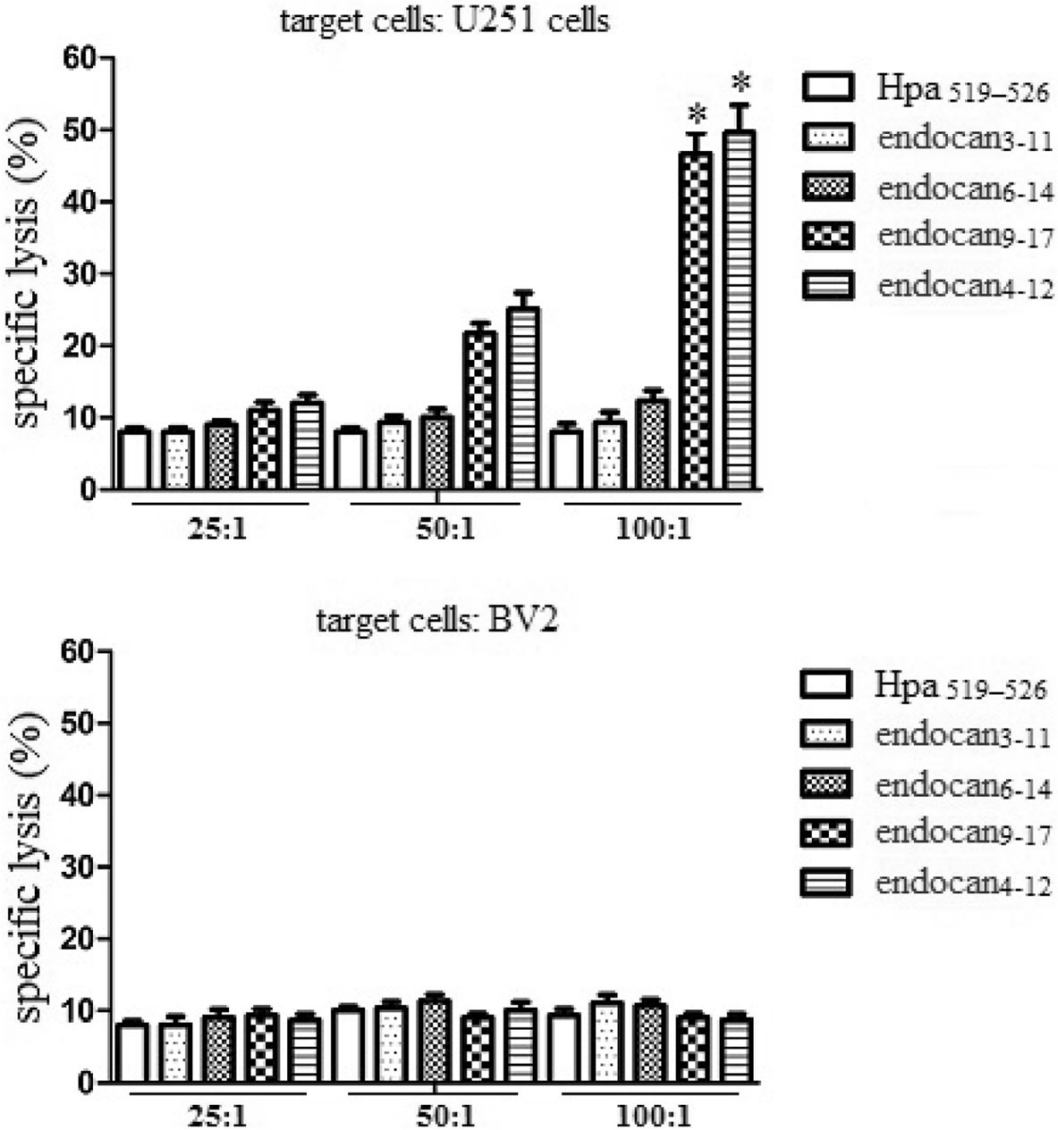
Specific lysis of endocan-derived peptide elicited CTLs. The PBMCs of human HLA-A2+ donors were obtained and then cultured in RPMI 1640 supplemented with 10% FCS, 100 U/ml penicillin, and 100 μg/ml streptomycin. DCs were generated, and loaded with different peptides at a final concentration of 100 μg/ml for 4 h and were then irradiated with 20 Gy, which prevented all outgrowths in the control cultures. Autologous T cells were restimulated every 7 days with the peptide-pulsed DCs to generate peptide-specific CTLs. Target cells were incubated with ^51^ Cr (100 μCi per 1× 10^6^ cells; Amersham Biosciences Corp) for 2 h in a 37 °C water bath. After incubation with ^51^Cr, target cells were washed three times with PBS, resuspended in RPMI 1640, and mixed with effector cells at effector-to-target (E/T) ratios of 25:1, 50:1, or 100:1. The percentage of cytotoxicity was calculated as follows: percentage of lysis = (sample cpm- spontaneous cpm)/(maximum cpm- spontaneous cpm) × 100%. Experiments performed in triplicate showed consistent results. Compared with control, *P < 0.05

To detect whether predicted peptides could elicit CTL to lyze target cells, we utilized the peptides to elicit PBMCs from HLA-A2.1+ donors. As shown in Fig. 3, endocan_**4–12**_ and endocan_**9–17**_ could elicit specific CTL to lyse target cells expressing endocan and HLA-A2.1. However, the lysate could not be detected in endocan_**6–14**_ and endocan_**3–11**_ groups. These results suggested that endocan_**4–12**_ and endocan_**9–17**_ could effectively elicit CTL mediated cytotoxicity.

### Antibody inhibition assay

**Fig. 4:**
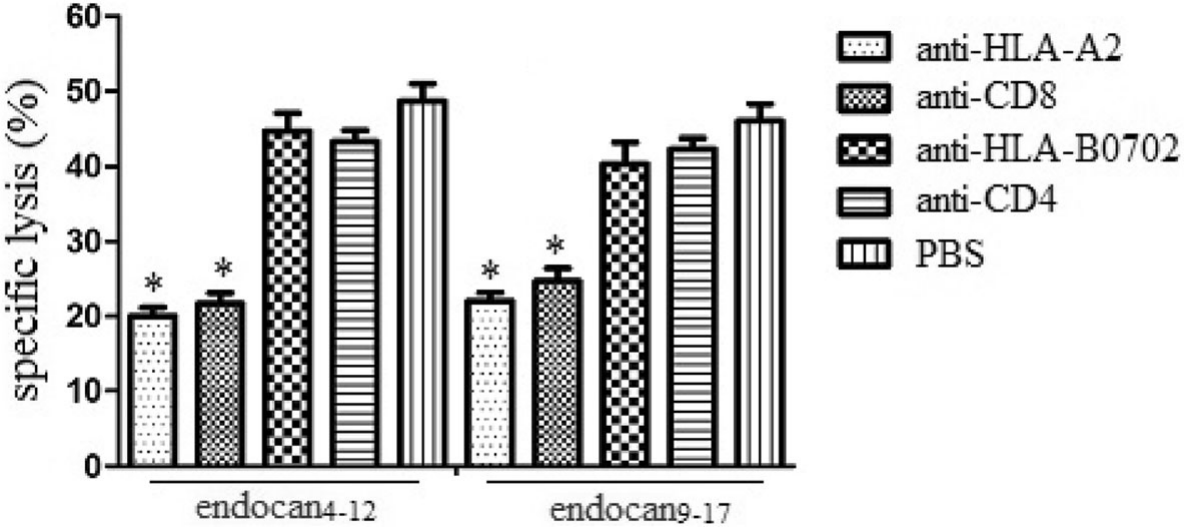
Antibody inhibition assay by anti-HLA-A2 or anti-CD8 antibody. Peptide-coated T2 target cells were incubated with or without anti-HLA-A2 antibody from BB7.2 cells for 1 h at 4 °C. Moreover, effectors induced by different endocan -derived peptides were also incubated with or without anti-CD8 antibody for 1 h at 4 °C. The cytotoxic activities of CTLs against T2 cells were analyzed at various E/T ratios by 51 Cr release assay. Experiments performed in triplicate showed consistent results. Compared with control, *P < 0.05

To further identify whether peptide induced effectors recognized target cells in an HLA-A2-restricted manner and whether the effectors were CD8+ T lymphocytes, HLA-A2 mAbs and CD8 mAbs were utilized to block recognition of target cells and effectors. In addition, HLA-B0702 mAbs and CD4 mAbs were served as negative controls. As Fig. 4 demonstrated, cytotoxicity of endocan_**4–12**_ and endocan_**9–17**_ specific CTLs was significantly attenuated after blocking by HLA-A2 mAbs and CD8 mAbs. However, cytotoxicity of endocan-specific CTLs could not been attenuated by negative controls. These results indicated that peptide induced effectors were HLA-A2.1- restricted and were mainly from CD8+ T lymphocytes.

### Induction of epitope-specific CTLs in vivo

**Fig. 5:**
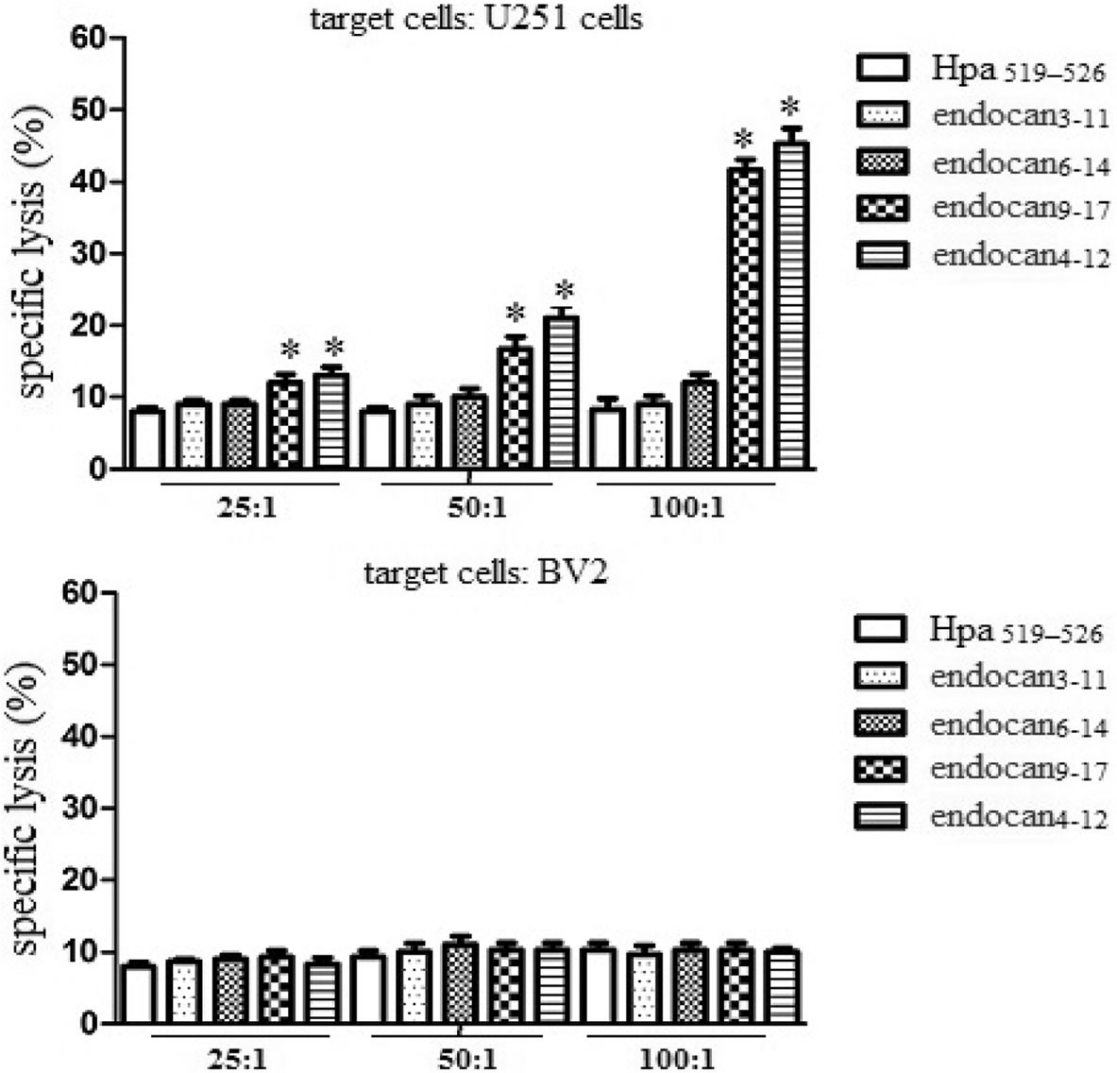
In vivo induction of epitope-specific CTLs in vivo. 12 weeks old HLA-A*0201/Kb mice were immunized three times once a week, by subcutaneous injection in the back with 1 × 10^6^ syngeneic mature DC pulsed with the peptides. After 7 days, the spleens of the mice were removed and the splenocytes were harvested as effectors. The cytotoxic activities of CTLs were determined against target cells at various E/T ratios using 51 Cr release assay. Experiments performed in triplicate showed consistent results. Compared with controls, *P < 0.05

Furthermore, we investigated whether peptide could induce immune response in vivo. HLA-A*0201/Kb mice were immunized by subcutaneous injection with syngeneic mature DC pulsed with the peptides. After 7 days, the splenocytes were harvested as effectors to analyze the cytotoxicity. As Fig. 5 demonstrated, endocan_**4–12**_ and endocan_**9–17**_ could induce CTL response to lyse endocan and HLA-A2.1 positive cells with high efficiency. These results suggested that the peptides also had immunogenicity in vivo.

## Discussion

Glioma is a fatal disease with median survival of 1.5 years [21]. Despite decades of therapeutial strategies in developing surgical and radiation techniques and chemotherapy agents, the survival of patients with glioma remains limited [22–24].

The challenge in treating glioma is the rapid proliferation of the tumor cells, the diffuse infiltrative nature of the disease, and resistant clones during treatment [25–27].

Recently, the immune system has been proved to recognize and eliminate the spontaneous development of glioma cells [28–30]. Because of its potential to manipulate and attack tumor cells, immunotherapy represents promising strategy in the clinic [31–33]. The related strategy to regulate immune system includes checkpoint inhibitors, gene vector, oncolytic virus and vaccine therapy [34–36]. Of the treatment strategies, tumor-associated antigens (TAAs) of inducing a specific host immune response to tumors are particularly appealing because of high specificity for tumor cells and low toxicity to normal tissue [37–39].

DCs are regarded as the professional antigen-presenting cell that specializes in the initiating of antigen-specific CD8+ T cells [40]. DCs carry exogenous tumor antigens into endosomes and/or phagolysosomes and translocate these into the cytosol [41]. Furthermore, tumor antigens are delivered into the class I MHC processing pathway for presentation to tumor-specific CD8+ T cells [42]. Therefore, DCs loaded with TAAs represent ideal strategy for tumor antigen vaccination.

Endocan is a secreted proteoglycan that is upregulated by growth factors and chemokines in vitro and on tumor vasculature in various cancers [43, 44]. Endocan can be upregulated by angiogenic factors and inflammatory cytokines, such as tumor necrosis factor-α and interleukin-1β [45]. In tumor tissue sections, a stromal inflammatory reaction was observed only in tumors overexpressing endocan polypeptide, and depletion of CD122+ cells was able to delete partially the anti-tumor effect of endocan polypeptide. These results reveal a novel pathway for endocan in the control of tumor growth, which involves inflammatory cells of the innate immunity [44]. In addition, an increase in tissue expression or serum level of endocan reflects endothelial activation and neovascularization which are prominent pathophysiological changes associated with inflammation and tumor progression. Consequently, enedocan has been used as a blood-based and tissue-based biomarker for various cancers and inflammation and has shown promising results [46].

In previous studies, endocan was reported to induce tumor formation and progression [11]. Recently, endocan has been identified to be overexpressed at the messenger RNA and/or protein levels in several tumor types, including glioblastoma, nonsmall cell lung cancer, gastric cancer, and hepatocellular carcinoma [13, 47, 48]. Most evidence suggested that endocan overexpression contributed to aggressive tumor progression and poor outcomes [9, 49, 50].

In current experiment, four candidate epitopes with highest scores from endocan were predicted according to HLA-A2.1-restricted epitope prediction algorithms. Secondly, the affinity of each epitope with HLA-A2.1 was analyzed by peptide binding assay. The data demonstrated that endocan_**4–12**_ and endocan_**9–17**_ showed high affinity to HLA-A2.1 molecule. Thirdly, the activity of epitope specific CTLs was measured by ELISPOT and ^51^Cr release assay. The results demonstrated that endocan_**4–12**_ and endocan_**9–17**_ could elicit IFN-γ-producing cells and lyse target cells in an HLA-A2.1-restricted manner. Lastly, HLA-A*0201/Kb mice were immunized with predicted peptides, and the specific CTLs elicited in vivo were analyzed. The results demonstrated that endocan_**4–12**_ and endocan_**9–17**_ could also generate CTLs to lyse endocan and HLA-A2.1 positive target cells. These results suggested that the peptides also had immunogenicity in vivo.

## Conclusions

Taken together, our results suggest that endocan_**4–12**_ and endocan_**9–17**_ could elicit specific HLA-A2.1-restricted CTLs and exert antitumor potential. In addition, the novel epitopes can be acted as a promising tumor vaccine, and may represent a new strategy for tumor immunotherapy in the clinic.

## Data Availability

Please contact author for data requests.

## References

[1] Lam FC, Morton SW, Wyckoff J, Vu Han TL, Hwang MK (2018). Enhanced efficacy of combined temozolomide and bromodomain inhibitor therapy for gliomas using targeted nanoparticles. Nat Commun.

[2] Filbin MG, Tirosh I, Hovestadt V, Shaw ML, Escalante LE (2018). Developmental and oncogenic programs in H3K27M gliomas dissected by single-cell RNA-seq. Science.

[3] Komori T, Muragaki Y, Chernov MF (2018). Pathology and genetics of Gliomas. Prog Neurol Surg.

[4] Wong ET, Lok E, Swanson KD (2018). Alternating electric fields therapy for malignant Gliomas: from bench observation to clinical reality. Prog Neurol Surg.

[5] Paldor I, Chaichana KL, Brem H, Tyler BM (2018). Targeted local therapy for Management of Intracranial High-Grade Gliomas. Prog Neurol Surg.

[6] Parker Kerrigan BC, Hossain A, Yamashita S, Lang FF (2018). Stem cell therapy of Gliomas. Prog Neurol Surg.

[7] Qiu CR, Fu Q, Sui J, Zhang Q, Wei P (2017). Endocan: endothelial dysfunction, inflammation, or both?. Angiology.

[8] Emet S, Elitok A, Onur I, Kocaaga M, Bilge AK (2017). Endocan: a novel biomarker associated with well-developed coronary collateral circulation in patients with stable angina and chronic total occlusion. J Thromb Thrombolysis.

[9] Sumei Z, Shaolong C, Xiang W, Yinliang Q, Qing Z (2016). Endocan reduces the malign grade of gastric cancer cells by regulating associated protein expression. Tumour Biol.

[10] Cornelius A, Cortet-Rudelli C, Assaker R, Kerdraon O, Gevaert MH (2012). Endothelial expression of endocan is strongly associated with tumor progression in pituitary adenoma. Brain Pathol.

[11] Roudnicky F, Poyet C, Wild P, Krampitz S, Negrini F (2013). Endocan is upregulated on tumor vessels in invasive bladder cancer where it mediates VEGF-A-induced angiogenesis. Cancer Res.

[12] Sagara A, Igarashi K, Otsuka M, Kodama A, Yamashita M (2017). Endocan as a prognostic biomarker of triple-negative breast cancer. Breast Cancer Res Treat.

[13] Huang X, Chen C, Wang X, Zhang JY, Ren BH (2016). Prognostic value of endocan expression in cancers: evidence from meta-analysis. Onco Targets Ther.

[14] Yang J, Sheng S, Yang Q, Li L, Qin S (2017). Endocan silencing induces programmed cell death in hepatocarcinoma. Oncol Lett.

[15] Mertoglu C, Gunay M, Yerligok O (2018). Could Endocan, a marker of inflammation and endothelial dysfunction, be a new diagnostic marker for fibromyalgia?. Clin Lab.

[16] Gaudet A, Portier L, Prin M, Copin MC, Tsicopoulos A (1985). (2019) Endocan regulates acute lung inflammation through control of leukocyte diapedesis. J Appl Physiol.

[17] Liu H, Chen L, Liu J, Meng H, Zhang R (2017). Co-delivery of tumor-derived exosomes with alpha-galactosylceramide on dendritic cell-based immunotherapy for glioblastoma. Cancer Lett.

[18] Saito H, Kitagawa K, Yoneda T, Fukui Y, Fujsawa M (2017). Combination of p53-DC vaccine and rAd-p53 gene therapy induced CTLs cytotoxic against p53-deleted human prostate cancer cells in vitro. Cancer Gene Ther.

[19] Choi CW, Jeong MH, Park YS, Son CH, Lee HR, et al. Combination treatment of stereotactic body radiation therapy and immature dendritic cell vaccination for augmentation of local and systemic effects. Cancer Res Treat. 2018. 10.4143/crt.2018.186 PMC6473298 29879758

[20] Lanterna C, Musumeci A, Raccosta L, Corna G, Moresco M (2016). The administration of drugs inhibiting cholesterol/oxysterol synthesis is safe and increases the efficacy of immunotherapeutic regimens in tumor-bearing mice. Cancer Immunol Immunother.

[21] Jo J, Wen PY (2018). Antiangiogenic therapy of high-grade Gliomas. Prog Neurol Surg.

[22] Wesseling P, Capper D (2018). WHO 2016 classification of gliomas. Neuropathol Appl Neurobiol.

[23] Mount CW, Majzner RG, Sundaresh S, Arnold EP, Kadapakkam M (2018). Potent antitumor efficacy of anti-GD2 CAR T cells in H3-K27M(+) diffuse midline gliomas. Nat Med.

[24] Zhou Y, Tan Z, Chen K, Wu W, Zhu J (2018). Overexpression of SHCBP1 promotes migration and invasion in gliomas by activating the NF-kappaB signaling pathway. Mol Carcinog.

[25] Ishikawa E, Muragaki Y, Yamamoto T, Ohno T, Matsumura A (2018). Vaccine therapy of high-grade Gliomas. Prog Neurol Surg.

[26] Iwami K, Natsume A, Wakabayashi T (2018). Cytokine therapy of Gliomas. Prog Neurol Surg.

[27] Butowski N (2018). Novel and prospective molecular targets for therapy of intracranial Gliomas. Prog Neurol Surg.

[28] Ciaglia E, Laezza C, Abate M, Pisanti S, Ranieri R (2018). Recognition by natural killer cells of N6-isopentenyladenosine-treated human glioma cell lines. Int J Cancer.

[29] Zhu C, Chrifi I, Mustafa D, van der Weiden M, Leenen PJM (2017). CECR1-mediated cross talk between macrophages and vascular mural cells promotes neovascularization in malignant glioma. Oncogene.

[30] Wildes TJ, Grippin A, Dyson KA, Wummer BM, Damiani DJ (2018). Cross-talk between T cells and hematopoietic stem cells during adoptive cellular therapy for malignant Glioma. Clin Cancer Res.

[31] Baker GJ, Chockley P, Zamler D, Castro MG, Lowenstein PR (2016). Natural killer cells require monocytic gr-1(+)/CD11b(+) myeloid cells to eradicate orthotopically engrafted glioma cells. Oncoimmunology.

[32] Chitadze G, Lettau M, Luecke S, Wang T, Janssen O (2016). NKG2D- and T-cell receptor-dependent lysis of malignant glioma cell lines by human gammadelta T cells: modulation by temozolomide and a disintegrin and metalloproteases 10 and 17 inhibitors. Oncoimmunology.

[33] Orozco-Morales M, Sanchez-Garcia FJ, Golan-Cancela I, Hernandez-Pedro N, Costoya JA (2015). RB mutation and RAS overexpression induce resistance to NK cell-mediated cytotoxicity in glioma cells. Cancer Cell Int.

[34] Qin K, Tian G, Li P, Chen Q, Zhang R (2012). Anti-glioma response of autologous T cells stimulated by autologous dendritic cells electrofused with CD133+ or CD133- glioma cells. J Neuroimmunol.

[35] Chen H, Yuan B, Zheng Z, Liu Z, Wang S (2011). A novel vaccine containing EphA2 epitope and LIGHT plasmid induces robust cellular immunity against glioma U251 cells. Cell Immunol.

[36] Adach-Kilon A, Swiatek-Machado K, Kaminska B, Dabrowski M (2011). Signal transducer and activator of transcription 1 (Stat1) maintains basal mRNA expression of pro-survival stat3-target genes in glioma C6 cells. J Cell Biochem.

[37] Liu Z, Poiret T, Persson O, Meng Q, Rane L (2018). NY-ESO-1- and survivin-specific T-cell responses in the peripheral blood from patients with glioma. Cancer Immunol Immunother.

[38] Liu Y, Yuelling LW, Wang Y, Du F, Gordon RE (2017). Astrocytes promote Medulloblastoma progression through hedgehog secretion. Cancer Res.

[39] Liu H, Smith AJ, Ball SS, Bao Y, Bowater RP (2017). Sulforaphane promotes ER stress, autophagy, and cell death: implications for cataract surgery. J Mol Med (Berl).

[40] van Kooyk Y, Unger WW, Fehres CM, Kalay H, Garcia-Vallejo JJ (2013). Glycan-based DC-SIGN targeting vaccines to enhance antigen cross-presentation. Mol Immunol.

[41] Eleveld-Trancikova D, Sanecka A, van Hout-Kuijer MA, Looman MW, Hendriks IA (2010). DC-STAMP interacts with ER-resident transcription factor LUMAN which becomes activated during DC maturation. Mol Immunol.

[42] Weng D, Calderwood SK, Gong J (2018). A novel heat shock protein 70-based vaccine prepared from DC-tumor fusion cells. Methods Mol Biol.

[43] Turer CC, Durmus D, Balli U, Guven B (2017). Effect of non-surgical periodontal treatment on gingival Crevicular fluid and serum Endocan, vascular endothelial growth factor-a, and tumor necrosis factor-alpha levels. J Periodontol.

[44] Yassine H, De Freitas CN, Depontieu F, Scherpereel A, Awad A (2015). The non glycanated endocan polypeptide slows tumor growth by inducing stromal inflammatory reaction. Oncotarget.

[45] Abid MR, Yi X, Yano K, Shih SC, Aird WC (2006). Vascular endocan is preferentially expressed in tumor endothelium. Microvasc Res.

[46] Kali A, Shetty KS (2014). Endocan: a novel circulating proteoglycan. Indian J Pharmacol.

[47] Yu PH, Chou SF, Chen CL, Hung H, Lai CY (2013). Upregulation of endocan by Epstein-Barr virus latent membrane protein 1 and its clinical significance in nasopharyngeal carcinoma. PLoS One.

[48] Matano F, Yoshida D, Ishii Y, Tahara S, Teramoto A (2014). Endocan, a new invasion and angiogenesis marker of pituitary adenomas. J Neuro-Oncol.

[49] Grigoriu BD, Depontieu F, Scherpereel A, Gourcerol D, Devos P (2006). Endocan expression and relationship with survival in human non-small cell lung cancer. Clin Cancer Res.

[50] Delehedde M, Devenyns L, Maurage CA, Vives RR (2013). Endocan in cancers: a lesson from a circulating dermatan sulfate proteoglycan. Int J Cell Biol.

